# Volatile Metabolites of Pathogens: A Systematic Review

**DOI:** 10.1371/journal.ppat.1003311

**Published:** 2013-05-09

**Authors:** Lieuwe D. J. Bos, Peter J. Sterk, Marcus J. Schultz

**Affiliations:** 1 Department of Intensive Care, Academic Medical Center, University of Amsterdam, Amsterdam, The Netherlands; 2 Department of Respiratory Medicine, Academic Medical Center, University of Amsterdam, Amsterdam, The Netherlands; 3 Laboratory of Experimental Intensive Care and Anesthesiology, Academic Medical Center, University of Amsterdam, Amsterdam, The Netherlands; International Centre for Genetic Engineering and Biotechnology, India

## Abstract

Ideally, invading bacteria are detected as early as possible in critically ill patients: the strain of morbific pathogens is identified rapidly, and antimicrobial sensitivity is known well before the start of new antimicrobial therapy. Bacteria have a distinct metabolism, part of which results in the production of bacteria-specific volatile organic compounds (VOCs), which might be used for diagnostic purposes. Volatile metabolites can be investigated directly in exhaled air, allowing for noninvasive monitoring. The aim of this review is to provide an overview of VOCs produced by the six most abundant and pathogenic bacteria in sepsis, including *Staphylococcus aureus*, *Streptococcus pneumoniae*, *Enterococcus faecalis*, *Pseudomonas aeruginosa*, *Klebsiella pneumoniae*, and *Escherichia coli*. Such VOCs could be used as biological markers in the diagnostic approach of critically ill patients. A systematic review of existing literature revealed 31 articles. All six bacteria of interest produce isopentanol, formaldehyde, methyl mercaptan, and trimethylamine. Since humans do not produce these VOCs, they could serve as biological markers for presence of these pathogens. The following volatile biomarkers were found for identification of specific strains: isovaleric acid and 2-methyl-butanal for *Staphylococcus aureus*; 1-undecene, 2,4-dimethyl-1-heptane, 2-butanone, 4-methyl-quinazoline, hydrogen cyanide, and methyl thiocyanide for *Pseudomonas aeruginosa;* and methanol, pentanol, ethyl acetate, and indole for *Escherichia coli*. Notably, several factors that may effect VOC production were not controlled for, including used culture media, bacterial growth phase, and genomic variation within bacterial strains. In conclusion, VOCs produced by bacteria may serve as biological markers for their presence. Goal-targeted studies should be performed to identify potential sets of volatile biological markers and evaluate the diagnostic accuracy of these markers in critically ill patients.

## Introduction

Sepsis is increasingly prevalent in the developed world, affecting 240 per 100,000 persons per year [1]. Early start of targeted antibiotics lowers mortality [2]. However, in the majority of cases, empirical antibiotic treatment is untargeted due to inadequate diagnostics, resulting in a three-fold increase in mortality when compared to targeted antibiotic treatment [3].

Ideally, invasion of morbific pathogens is detected as early as possible; the strain of the causative pathogens is identified swiftly, and antimicrobial sensitivity is rapidly known, preferably before start of antimicrobial therapy. However, cultures may take days to become positive and have limited sensitivity, especially in patients already receiving antibiotics because of a previous infection [4]. In addition, contamination could lead to false-positive results and therefore may increase prescription of unnecessary antibiotics [5]. Gram-stain results and direct cellular examination (e.g., of bronchoalveolar lavage fluid) are rapidly available but have limited sensitivity and specificity, and neither tell the exact strain of pathogen nor its antimicrobial sensitivity [6], [7]. These disadvantages also apply to several biomarkers (C-reactive protein, procalcitonine, pro-adrenomedullin and endotoxin) 8–10. PCR-based diagnostics are currently under investigation and although the results are promising, PCR takes hours before results are available and is laborious and costly [11].

In ancient times, physicians relied heavily on their senses before sophisticated analytical techniques became available. Color, taste, and smell were used to detect biological markers [12]. Bacteria are known to have characteristic smells. Bacterial strains have a distinct metabolism, part of which results in the production of bacteria-specific volatile organic compounds (VOCs) [13]–[15]. The metabolic pathways have been described for bacteria in several excellent review articles [14], [15]. However, reviews have not yet focused on pathogens and clinical problems.

Detection and identification of VOCs using sophisticated technology may have diagnostic value in medicine [14], [16], [17]. These techniques include gas chromatography and mass spectrometry (GC-MS), selected ion flow tube mass spectrometry (SIFT-MS) [18], ion-molecule reaction mass spectrometry (IMR-MS) [19], [20], and electronic noses (eNoses) [16], [21]. GC-MS is used as a gold standard for separation, detection, and identification of VOCs. SIFT-MS and IMR-MS allow for real-time measurement of some VOCs. eNoses do not identify VOCs but rely on pattern recognition [16]. Volatile compounds can be investigated in vitro (in culture media or directly in patient material) or directly in the exhaled air (in vivo), allowing for noninvasive monitoring.

Three goals could be pursued with VOC detection: (1) proof absence of bacterial pathogens (i.e., very high sensitivity and negative predictive value, and therefore no start of antibiotic treatment), (2) identify the presence of a specific strain of bacteria (i.e., very high specificity and positive predictive value, and thus start of appropriate antimicrobial therapy), and (3) separation between phenotypes within bacterial species and therefore prevention of start of antibiotics to which the causative pathogens are not sensitive. However, before VOCs can be tested for these goals in clinical trials, possible diagnostic targets per goal should be known. Therefore, the aim of this review is to provide an overview of volatile organic compounds produced by the six most abundant and pathogenic bacteria in sepsis: *Staphylococcus aureus* (SA), *Streptococcus pneumoniae* (SP), *Enterococcus faecalis* (EF), *Pseudomonas aeruginosa* (PA), *Klebsiella pneumoniae* (KP) and *Escherichia coli* (EC) [22].

## Results

**Figure 1:**
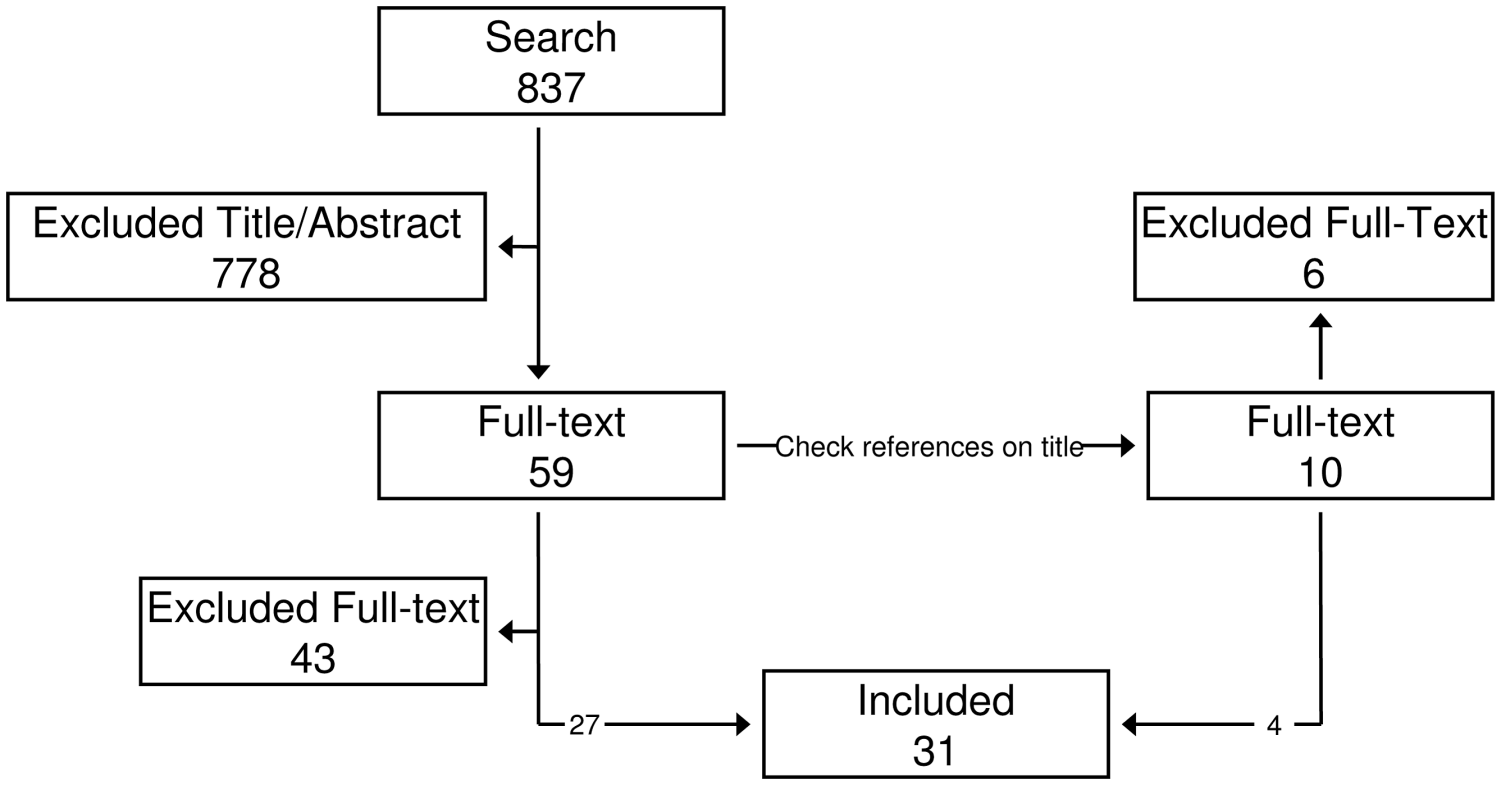
Inclusion flow diagram. The initial search resulted in 837 hits. Fifty-nine were selected based on title and abstract. Full text was read and references were checked for additional hits. This resulted in ten additional hits. Thirty papers were included based on the full text.

The MEDLINE search resulted in 837 articles of which 778 were excluded based on title and/or abstract (Figure 1). Fifty-nine articles were read and tested for inclusion criteria. This resulted in the inclusion of twenty-seven articles (Table 1). Ten articles were read based on references, of which four were included, bringing the sum of included articles to thirty–one.

**Table 1:** Literature.

Year	1^st^ Author	Pathogen	Method	Remarks	Reference
1977	Hayward	SA, PA, EC	GLC	Through references	[65]
1979	Cox	PA	GC + Colorimetric	Through references	[66]
1980	Labows	PA	GC-MS	Pathway description	[39]
1984	Davies	SA, PA, EC	HS-GLC		[67]
1986	Zechman	SA, PA, KP	GC-MS		[28]
1995	Kuzma	PA, EC	GC-MS	Pathway description	[24]
1997	Scholler	PA	GC-FID		[68]
2000	Julák	SA, SP, EF, PA, KP, EC	GC-MS		[69]
2003	Julák	SA, SP, EF, PA, KP, EC	GC-FID	Clinical samples	[53]
2005	Carroll	PA	SIFT-MS	Clinical samples	[70]
2005	Hamilton-Kemp	EC	GC-MS	Through references	[71]
2006a	Allardyce	SA, SP, PA, EC	SIFT-MS	Antibiotic effects	[56]
2006b	Allardyce	SA, SP, PA, EC	SIFT-MS	Two different timepoints	[51]
2006	Julak	PA	SIFT-MS	Clinical samples	[52]
2006	Scotter	SA, SP, PA, EC	SIFT-MS		[72]
2008	Bunge	EC	PTR-MS	Different timepoints	[73]
2008	Syhre	SA, SP, EC	GC-MS	Clinical samples	[35]
2009	Maddula	EC	MCC-IMS + GC-MS		[74]
2009	Preti	SA, PA	GC-MS	Clinical samples	[54]
2010	Scott-Thomas	PA	GC-MS	Clinical samples	[40]
2010	Thorn	SA, EF, PA, EC	SIFT-MS	Multivariate analysis	[13]
2010	Zhu	SA, PA EC	SESI-MS		[75]
2010	Chambers	SP	GC-MS		[34]
2011	Savelev	PA	GC-MS	Clinical samples	[60]
2011	Shestivska	PA	GC-MS		[76]
2011	Storer	SA, EF, PA, KP, EC	SIFT-MS	Inoculated urine	[77]
2012	Bean	PA	GC/GC-TOF-MS		[78]
2012a	Dolch	PA, EC	IMR-MS	Two different timepoints	[49]
2012b	Dolch	SA, EF	IMR-MS	Two different timepoints	[50]
2012	Filipiak	SA, PA	GC-MS	Different timepoints	[48]
2012	Junger	SA, SP, PA, KP, EC	MCC-IMS + GC-MS		[79]

*SA = Staphylococcus aureus, SP = Streptococcus pneumoniae, EF = Enterococcus faecalis, PA = Pseudomonas aeruginosa, KP = Klebsiella pneumoniae, EC = Escherichia coli.*

**Figure 2:**
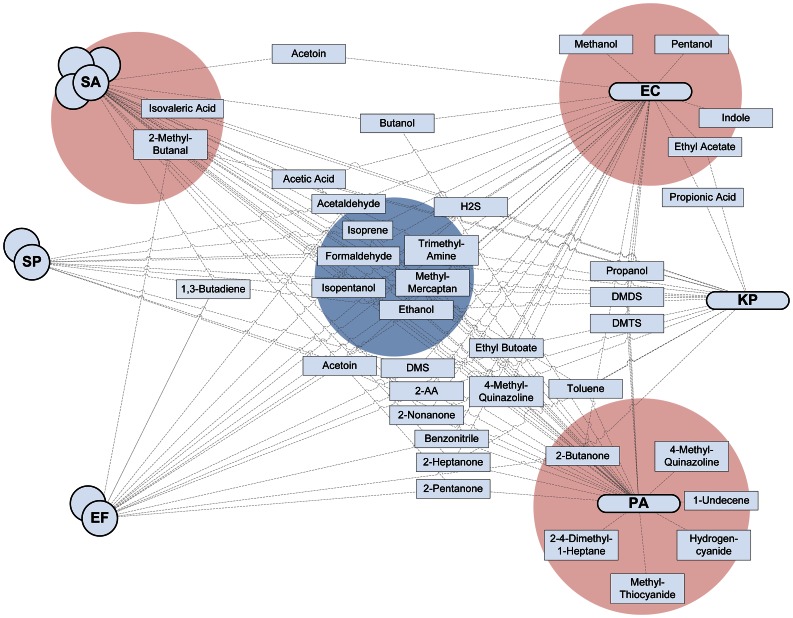
Interaction plot. The six investigated pathogenic bacteria are plotted on both sides, with gram-positive bacteria on the left and gram-negative on the right. All the metabolites for which convincing evidence on production by at least one of the bacteria was available (as indicated by a green cell in Tables S1 to S9 in Text S1) were included in the figure and connected with a line to all bacteria known to produce a particular metabolite. The stronger the available evidence for the production of a metabolite by one strain of bacteria, the closer the metabolite is situated to the pathogen. Four zones of interest are highlighted. The blue zone in the middle indicates metabolites that are (almost) always produced by all pathogens and are therefore candidate markers with a high sensitivity that might thus qualify for the exclusion of infection (high negative predictive value). The three red zones indicate metabolites that are produced by only or mainly one strain of bacteria; these are possibly volatile biomarkers specific for a pathogen with a very high positive predictive value.

The articles originated from 1977 to 2012, with a rapid increase in the number of publications since 2006. Fifteen articles reported on data collected with GC-MS, seven on data collected with SIFT-MS, three with IMR-MS, and six with other techniques. Seven studies used clinical samples; twenty studies used reference strains. Results on 161 metabolites were obtained, of which a minority was studied in multiple papers. The findings are reported in Tables S1 to S9 in Text S1 and summarized below, per functional group. The most prominent VOCs and their (cross-) association with the six selected gram-positive and gram-negative bacteria are illustrated in Figure 2.

### Hydrocarbons (Table S1 in Text S1)

In Table S1 in Text S1, the hydrocarbons investigated in prespecified pathogens are listed. One of the most investigated hydrocarbons is isoprene **[#13]**, which seems to be produced in both gram-positive and gram-negative bacteria, although studies show contradicting results on their presence. The production is most likely through the methylerythritol phosphate pathway, is growth dependent (high during the log phase and low during the stationary phase), and occurs primarily in a nutrient-rich environment [23], [24]. Isoprene is also one of the main volatiles in the breath of mammals and is less applicable as a focus for in vivo studies [25]. 1-undecene **[#6]** and other less well-studied alkenes **[#3–5]** are suggested to be produced mainly by PA, and are most likely the product of degradation of fatty acids through the b-oxidation pathway, a pathway that is suggested for most volatile hydrocarbons [15], [26]. 1,3-butadiene **[#2]** is reported to be produced by gram-positive bacteria, but not by gram-negative bacteria.

### Alcohols (Table S2 in Text S1)

1-alcohols are produced through b- or a-oxidation of fatty acid derivates through acetyl-CoA. Ethanol **[#30]** is one of the most studied volatiles [15]. It can be produced by all investigated bacteria, but some (SA, SP, KP, and EC) produce it almost always, while others (EF and PA) have been found to lack ethanol in the headspace. Methanol **[#32]**, propanol **[#34]**, butanol **[#27]**, pentanol **[#36]**, and some longer chain 1-alcohols **[#28, 29, 35]** are most prominently produced by EC, however not exclusively. EC might use these alcohols to inhibit the growth of other bacteria [27]. The branched alcohol isopentanol **[#25]** is found less frequently in EC, compared to the other pathogens. This metabolite is produced through another pathway, possibly via isovaleryl-CoA, since concentrations increase when leucine is added to the growth medium [28].

### Acids (Table S3 in Text S1)

Fatty acids could be a marker for anaerobic metabolism, but are not strain specific [29], [30]. However, anaerobic-dependent production does not apply to very short, volatile fatty acids. Acetic acid **[#37]** is most frequently produced by SA, but also by the other pathogens. Isovaleric acid **[#42]** is more exclusively produced by SA. Propionic acid **[#44]** has only been reported in the headspace of KP. Other acids **[#38–41, 45]** have not been identified in the headspace of the pathogens studied in that review.

### Aldehydes (Table S4 in Text S1)

Formaldehyde **[#56]** is produced by many bacteria [31], including the six species on which we focus in this review. Acetaldehyde **[#54]** is also produced by most pathogens, but PA and KP are less likely to produce this metabolite. Notably, acetaldehyde and benzaldehyde **[#55]** are known to have antimicrobial activity [32]. Methylpropanal **[#49]**, 3-methyl-butanal **[#53]**, and 2-methyl-butanal **[#48]** are modifications of amino acids and intermediates for the formation of many ester and branched ketones [15]. Methylpropanal and 2-methyl-butanal are mostly produced by SA, while methylpropanal is produced by all investigated pathogens.

### Ketones (Table S5 in Text S1)

Methyl ketones are produced during decarboxylation of fatty acid derivates. The smallest, acetone **[#81]**, is produced by most bacteria, but not under all circumstances. Furthermore, acetone is also present in high concentrations in breath, limiting the in vivo applicability as a biomarker for bacterial presence. The longer 2-ketones **[#62–70]** are classically biomarkers for PA [28], but the pooled results provide evidence only for 2-nonanone, 2-dodecanone, 2-pentanone, and 2-heptanone. 2-nonanone is also produced by SA. Acetoin or 3-hydroxybutanone **[#79]** is used to differentiate between lactose-fermenting and nonfermenting Enterobacteriaceae. Surprisingly, in one study, acetoin was found in the headspace of the nonfermenting EC. This suggests the involvement of other pathways for the production of acetoin [33]. In SA, acetoin generation has been linked to murein hydrolase activity, stationary-phase survival, and antibiotic resistance [33].

### Cyclic Compounds (Table S6 in Text S1)

2-phenylethanol **[#87]** is one of the most widespread microbial VOCs [15], but not in the hereby investigated pathogens. 2-pentylfuran **[#86]** has been proposed as a biomarker for *Aspergillus* [34], [35] but was also found in the headspace of SP. Two pathways of production via linoleic acid have been proposed: enzymatically controlled oxidation and direct interaction with reactive oxygen species [34]. Limonene **[#89]**, phenol **[#92]**, and toluene **[#93]** are identified as potential markers for bacterial presence in this study. This might imply that earlier statements that these compounds should be regarded as exogenous when found in a patient's breath need reconsideration [36]. In a study by Holland, germfree rats were compared to conventional rats [37]. Urinary acetophenone **[#88]** was increased 13-fold in conventional rats, suggesting bacterial production of this compound. This was indeed reported in one in vitro study. Several other compounds were found in conventional rats of which 4-heptanone **[#76]**, 2-heptanone **[#66]**, and 5-methyl-2-hexenal were the most significant and were also produced by bacteria in vitro.

### Esters (Table S7 in Text S1)

Ethyl acetate **[#100]** and other acetate-containing esters **[#99, 108]** are the product of esterification between acetic acid and a fatty acid. However, the pathogens producing most acetic acid, such as SA, are not the same as the ones with the most prominent ethyl acetate production, such as EC. The factors influencing this reaction remain unknown. Ethyl butanoate can be produced by all six pathogens, but is mostly found in *Enterococcus* and EC.

### S-Containing (Table S8 in Text S1)

The most important volatile sulfur-containing organic compounds are hydrogen sulfide **[#120]**, methyl mercaptan **[#122]**, dimethyl sulfide **[#118]**, dimethyl disulfide **[#117]**, and dimethyl trisulfide **[#119]**. All are highly toxic and might be involved in the induction of inflammation [38]. All bacteria are able to produce these compounds, but they might provide additional information about the species of pathogen. Hydrogen sulfide is produced mainly by EF and EC, while dimethyl disulfide is more frequently found in the gram-negative bacteria. Dimethyl trisulfide might be a marker for PA and dimethyl sulfide for PA and SP.

### N-Containing (Table S9 in Text S1)

The simplest N-containing volatile organic compound, ammonia **[#142]**, is most frequently produced by SA and PA. Hydrogen cyanide **[#147]** is only investigated in cultures of Pseudomonas, but was found to be produced in all studies. Trimethylamine **[#161]** might be a marker for PA and EC. 2-aminoacetophenone (2-AA) **[#130]** was recently proposed as a breath marker for PA and is responsible for the grape-like odor associated with PA infections [39], [40]. However, review of the literature clearly shows it can be produced by most bacteria and is frequently found in SP and EC. Furthermore, 2-AA can be found in a variety of food products and, after consumption, found in exhaled breath, resulting in false-positive results [41]. Indole **[#148]** is a direct product of deaminating L-tryptophan by tryptophanase. It is mainly produced by EC, but it is sporadically detectable in the headspace of other pathogens. Tryptophanase is essential for biofilm formation, thus indole can be regarded as a biomarker for this bacterial phenotype [42], [43].

## Discussion

Pathogenic bacteria are capable of producing a large variety of volatile metabolites. Our systematic review identified thirty-one articles reporting on VOC production by the most important pathogens of sepsis. However, only a very small fraction of the metabolites is produced exclusively by one of the bacterial species of interest. Notably, some studies failed to replicate the results of previous experiments, resulting in contradicting overall results. Despite these limitations, several sensitive and some very specific candidate biomarkers were identified by systematically summarizing the available literature (Figure 2).

The large number of contradicting results between the studies might be explained by four variables. Firstly, not all studies used the exact same subtype of bacterial species. In one study, phage types of SA influenced headspace volatile organic compounds [44]. Genomic variation between subtypes could result in differences in efficacy of enzymes within a specific metabolic pathway. These variations might be useful to phenotype within species of bacteria, though. However, this could hamper the clinical applicability of volatile biomarkers for strain identification. Secondly, the growth medium is the source of building blocks for the produced VOCs and therefore a confounding variable [44]–[47]. Thirdly, measurements were obtained at different timepoints in the growth of bacteria. Several studies investigated this phenomenon and found that depletion of metabolites and growth phase (log or stationary) influence headspace metabolites [48]–[51]. Lastly, the majority of the included studies investigated cultures of reference strains. However some studies focused on clinical samples, in which within-class variation was increased [52]–[54]. Patient samples are less well defined than laboratory-produced cultures of reference strains and are different in the following aspects: CFUs, growth phase, host response, viscosity [55], confounding comorbidities, and medications (e.g., antibiotics [56]).

Several biomarkers qualify for clinical investigation with regard to the first goal of biomarker research: proof absence of a bacterial pathogen. Isopentanol, formaldehyde, methyl mercaptan, and trimethylamine are produced by all bacteria and not by the host (blue area in Figure 2). Ethanol and isoprene are also sensitive candidate markers but are found in large quantities in the breath of mammals. If the aim of a study is to exclude bacterial infection from the differential diagnosis, a set of volatile biomarkers with a high a priori chance of being produced by a lot of pathogens should be investigated. Not finding any of these candidate markers might have a high negative predictive value.

Identification of specific strains might be performed using the following VOCs: SA – isovaleric acid and 2-methyl-butanal; PA – 1-undecene, 2,4-dimethyl-1-heptane, 2-butanone, 4-methyl-quinazoline, hydrogen cyanide, and methyl thiocyanide; EC – methanol, pentanol, ethyl acetate, and indole (red areas in Figure 2). No candidate biomarkers for SP, EF, and KP could be identified in the literature yet. For the identification of species of pathogens, a combination of volatile organic compounds is recommended. The advantages of this approach are illustrated in a recent paper by Thorn and in several studies using electronic nose technology [13], [55], [57], [58]. It is imperative that diagnostic accuracy (sensitivity and specificity) is reported following the STARD guidelines [59], as is only done in one of the studies included in this review [60].

Phenotyping of pathogens should focus on infecting/colonizing bacteria, bacterial growth, and bacterial resistance. Interestingly, indole was found to be a biomarker for biofilm formation in EC and might thus be used to separate between clinically relevant phenotypes within the same strain of bacteria. Secondly, small volatile sulfide-containing organic compounds were found to induce inflammation in a rat model and might thus serve as a marker for pathogenicity. Thirdly, the production of several VOCs is decreased after the addition of antibiotics in levels above MIC to the culture medium, suggesting that therapeutic response can be monitored. Antibiotic administration below MIC did lower VOC concentrations, but to a lower extent, suggesting a dose dependency. The influence of bacterial resistance on VOCs was not described in the included papers. However, the first steps in this direction are taken in a recent paper on colorimetric electronic nose technology discriminating methicillin-resistant SA from methicillin-sensitive SA and vancomycin-resistant EF from vancomycin-sensitive EF [58].

This review has several limitations. First of all, most included studies did not include all preselected pathogens and thus provide partial evidence for clinical questions involving all pathogens. Secondly, since most studies did not report quantitative measures and used different sampling techniques, no headspace concentrations could be given per compound per study. Thirdly, increased headspace concentrations were reported, but decreased concentrations may have been missed in some studies. Indeed, the absence of a normally present metabolite might be just as much proof of the presence of a pathogen as the presence of another VOC. Finally, different technologies were used to detect the volatile organic compounds. GC-MS was mostly used, although not always with the same materials and separation methods. However, while keeping the limitations of the used separation methods in mind, GC-MS remains the gold standard for volatile organic compound discovery. In this review, we also included studies using SIFT-MS, IMR-MS, and other techniques that allow for compound identification. These technologies are not as powerful as GC-MS in separating and identifying metabolites but were nevertheless included in this review since they did allow for identification of some compounds.

If volatile organic compounds are used in vivo for diagnostic purposes in sepsis, several considerations must be taken into account. First of all, the growth medium inside the host might be entirely different from in vitro growth media, resulting in a different set of produced metabolites. Secondly, the host will interact with the bacteria through an inflammatory response, which might alter metabolism. This inflammatory response can alter human metabolism in itself and future studies will need to address the metabolomic difference between and an infectious and a noninfectious inflammatory response [25], [61]. Thirdly, VOCs can be derived from diet and environment. Finally, the body, including the lungs, is host to a unique microbiome, even in healthy conditions [62], [63]. It might very well be that these residential bacteria produce similar metabolites and therefore interfere with a VOC-based diagnostic test. In this scenario, VOCs altered by inflammation might be used to further discriminate between colonizing and pathogenic bacteria.

In conclusion, several volatile biomarkers show to be particularly promising candidates for proof of absence of infection, whereas some others qualify for the detection of bacteria and identification of the six investigated bacterial species. However, only a limited amount of research is available. Therefore, targeted studies should be performed to identify potential sets of volatile biomarkers and evaluate the diagnostic accuracy of these markers in critically ill patients.

## Methods

A broad systematic search in the EMBASE library was performed on the August 1, 2012 using the following terms: “(mass and spectrometry) and bacteria and volatile”.

Articles were selected for full-text examination if the title and/or abstract suggested the investigation of bacterial pathogens in a clinically relevant setting and the measurement of volatile organic compounds.

Selected articles were read and included if (a) one or more of the following, most frequently cultured pathogens on the ICU [22], was investigated: *Staphylococcus aureus*, *Streptococcus pneumoniae*, *Enterococcus faecalis*, *Pseudomonas aeruginosa*, *Klebsiella pneumoniae*, or *Escherichia coli* and (b) a summary of detected volatile organic compounds per pathogen was provided. Furthermore, all references of the selected articles were scanned based on title and selected based on the previous criteria. Double publications of the same data were disregarded.

All volatile organic compounds described in the included articles were summarized in nine tables (see supplemental information: Tables S1, S2, S3, S4, S5, S6, S7, S8, S9 in Text S1) based on the following molecular structures (adapted from Hakim et al. [64]): hydrocarbons, alcohols, acids, aldehydes, ketones, cyclic compounds, esters, S-containing, and N-containing. They were referred to in the text by number **(#)**. If a molecule could be included in more than one table, the most appropriate category was chosen to avoid duplicates.

The production of a VOC by a pathogen in an article was indicated with a “+” and the absence of a molecule with a “–.” The Results section focuses on metabolites found in more than one study. The rows of these metabolites also received a coloring in Tables S1, S2, S3, S4, S5, S6, S7, S8, S9 in Text S1 based on level of evidence for absence or presence of a metabolite. Cells were colored based on the pooled results for a VOC per pathogen, for all included studies. A clear cell indicated there is little evidence (zero or one study). When there is convincing evidence a VOC is produced by a pathogen, the cell is colored green (more positive than negative evidence, with more than one study difference). A red cell means that pathogen is not known or rarely found to produce that molecule (more negative than positive evidence, with more than one study difference). Contradicting evidence resulted in an orange cell.

## Supporting Information

Text S1 Tables S1, S2, S3, S4, S5, S6, S7, S8, S9. Table S1: Volatile hydrocarbons produced by six pathogenic bacteria. Table S2: Volatile alcohols produced by six pathogenic bacteria. Table S3: Volatile acids produced by six pathogenic bacteria. Table S4: Volatile aldehydes produced by six pathogenic bacteria. Table S5: Volatile ketones produced by six pathogenic bacteria. Table S6: Cyclic volatile molecules produced by six pathogenic bacteria. Table S7: Volatile esters produced by six pathogenic bacteria. Table S8: S-containing volatile molecules produced by six pathogenic bacteria. Table S9: N-containing volatile molecules produced by six pathogenic bacteria.
(DOC)
