# Supplementary material for: Volatile Metabolites of Pathogens: A Systematic Review
Source: PLoS Pathog. 2013 May 9;9(5):e1003311. doi: 10.1371/journal.ppat.1003311 (PMC3649982; doi:10.1371/journal.ppat.1003311)
Supplement: Text S1 — Tables S1, S2, S3, S4, S5, S6, S7, S8, S9. Table S1: Volatile hydrocarbons produced by six pathogenic bacteria. Table S2: Volatile alcohols produced by six pathogenic bacteria. Table S3: Volatile acids produced by six pathogenic bacteria. Table S4: Volatile aldehydes produced by six pathogenic bacteria. Table S5: Volatile ketones produced by six pathogenic bacteria. Table S6: Cyclic volatile molecules produced by six pathogenic bacteria. Table S7: Volatile esters produced by six pathogenic bacteria. Table S8: S-containing volatile molecules produced by six pathogenic bacteria. Table S9: N-containing volatile molecules produced by six pathogenic bacteria. (DOC) [file ppat.1003311.s001.doc]

Supporting Information

Text S1

**Volatile metabolites of pathogens – a Systematic review**

Lieuwe DJ Bos, MSc^1,2,3^

Peter J Sterk, MD PhD^2^

Marcus J Schultz, MD PhD^1,3^

**Supplemental table – legend:**

SA = Staphylococcus areus, SP = Streptococcus pneumoniae, EF = Enterococcus faecalis, PA = Pseudomonas aeruginosa, KP = Klebsiella pneumoniae, EC = Escherichia coli

The production of a VOC by a pathogen in an article was indicated with a “+” and the absence of a molecule with a “−“. Cells were colored based on the pooled results for a VOC per pathogen, for all included studies. A clear cell indicated there is little evidence (zero or one study). When there is convincing evidence a VOC is produced by a pathogen, the cell is colored green (more positive than negative evidence, with more than one study difference). A red cell means that pathogen is not known or rarely found to produce that molecule (more negative than positive evidence, with more than one study difference). Contradicting evidence resulted in an orange cell.

Table S1: Volatile hydrocarbons produced by six pathogenic bacteria

| # |  | Structure | SA | SP | EF | PA | KP | EC | Remarks |
| --- | --- | --- | --- | --- | --- | --- | --- | --- | --- |
| **1** | 1,10−Undecadiene | [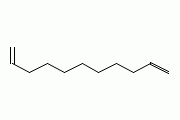](javascript:%20Enlargement('/image?db=1590&id=6192465&width=600&height=400',%20600,%20400);) |  |  |  | + [48] |  |  |  |
| **2** | 1,3−Butadiene | [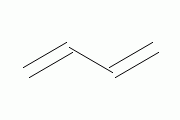](javascript:%20Enlargement('/image?db=1590&id=485905&width=600&height=400',%20600,%20400);) | − [50], + [48] |  | + [50], − [50] | − [49] |  | − [49] | Time dependent |
| **3** | 1−Decene | [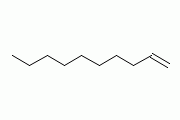](javascript:%20Enlargement('/image?db=1590&id=496595&width=600&height=400',%20600,%20400);) |  |  |  | + [48] |  |  |  |
| **4** | 1−Dodecene | [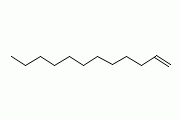](javascript:%20Enlargement('/image?db=1590&id=508348&width=600&height=400',%20600,%20400);) |  |  |  | + [48] |  |  |  |
| **5** | 1−Nonene | [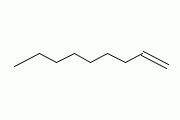](javascript:%20Enlargement('/image?db=1590&id=530002&width=600&height=400',%20600,%20400);) |  |  |  | + [48] |  |  |  |
| **6** | 1−Undecene | 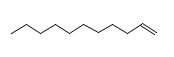 | − [28,79] | − [79] |  | + [13,28,48,68,79] | − [28,79] | − [79] |  |
| **7** | 10−Methyl−1−undecene | 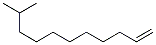 |  |  |  | + [48] |  |  |  |
| **8** | 2,4−Dimethyl−1−heptane | 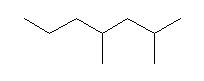 |  |  |  | + [7][60] |  |  |  |
| **9** | 2−Butene | [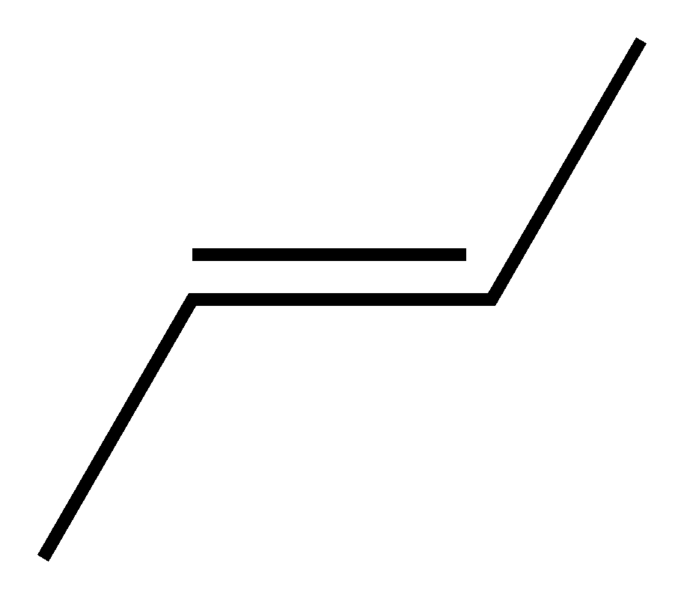](http://upload.wikimedia.org/wikipedia/commons/5/5a/Trans-but-2-ene-2D-skeletal.png) | + [48] |  |  |  |  |  |  |
| **10** | 2−Methyl−2−butene | [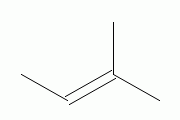](javascript:%20Enlargement('/image?db=1590&id=496366&width=600&height=400',%20600,%20400);) |  |  |  | + [48] |  |  |  |
| **11** | 2−Methyl−propene | [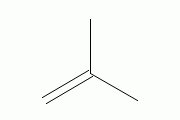](javascript:%20Enlargement('/image?db=1590&id=486086&width=600&height=400',%20600,%20400);) | + [48] |  |  |  |  |  |  |
| **12** | Hexane | [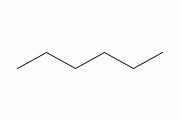](javascript:%20Enlargement('/image?db=1590&id=481432&width=600&height=400',%20600,%20400);) |  |  |  |  |  | + [74] |  |
| **13** | Isoprene | [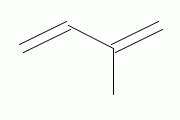](javascript:%20Enlargement('/image?db=1590&id=486128&width=600&height=400',%20600,%20400);) | − [13,50], + [50] |  | − [13,50], + [13,50] | + [13,24,48,68], − [49] |  | + [13,24], − [49] | Time dependent |
| **14** | Butane | [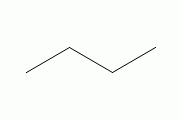](javascript:%20Enlargement('/image?db=1590&id=486805&width=600&height=400',%20600,%20400);) | + [48] |  |  | + [48] |  |  |  |
| **15** | Propane | [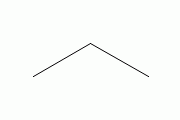](javascript:%20Enlargement('/image?db=1590&id=495596&width=600&height=400',%20600,%20400);) | + [48] |  |  |  |  |  |  |
| **16** | Propene | 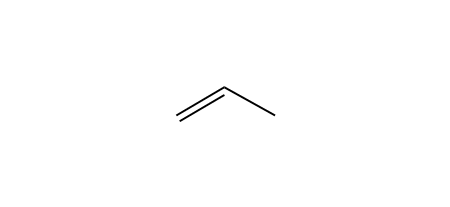 | − [72], + [50] | − [72] | + [50] | + [49,72], − [49] |  | − [49,72], + [49] | Time dependent |
| **17** | Undecane | [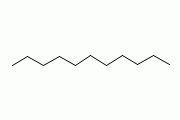](javascript:%20Enlargement('/image?db=1590&id=508532&width=600&height=400',%20600,%20400);) | − [28] |  |  | + [28] | + [28] |  |  |
| **18** | Undecene | 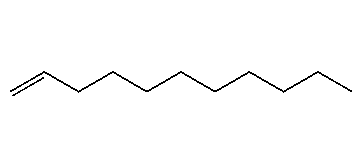 | − [28] |  |  | + [28] | − [28] |  |  |

Table S2: Volatile alcohols produced by six pathogenic bacteria

| **#** |  | Structure | SA | SP | EF | PA | KP | EC | Remarks |
| --- | --- | --- | --- | --- | --- | --- | --- | --- | --- |
| **19** | 1,2−Ethanediol | 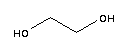 | + [75] |  |  | + [75] |  | − [75] |  |
| **20** | 2−Butanol | [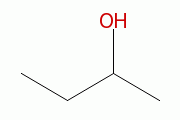](javascript:%20Enlargement('/image?db=1590&id=487263&width=600&height=400',%20600,%20400);) | + [28] |  |  | + [28,48] | + [28] |  |  |
| **21** | 2−Methyl−1−butanol | [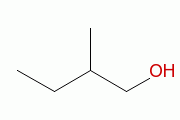](javascript:%20Enlargement('/image?db=1590&id=480741&width=600&height=400',%20600,%20400);) |  |  |  |  |  | + [73] |  |
| **22** | 2−Methyl−2−propanol | 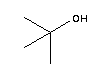 |  |  |  | + [78] |  |  |  |
| **23** | 2−Methyl−propanol | [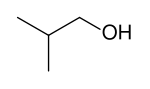](http://de.wikipedia.org/w/index.php?title=Datei:Isobutanol.svg&filetimestamp=20090203150852) | + [48] |  |  |  |  |  |  |
| **24** | 2−Pentanol | [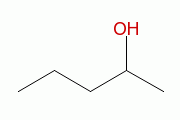](javascript:%20Enlargement('/image?db=1590&id=530523&width=600&height=400',%20600,%20400);) |  |  |  | + [78] |  |  |  |
| **25** | 3−Methyl−1−butanol = Isopentanol | [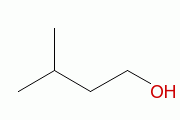](javascript:%20Enlargement('/image?db=1590&id=494081&width=600&height=400',%20600,%20400);) | + [28,48,75,79], − [51] | + [79], − [51] |  | + [28,39,60,78,79] , − [51,75] | + [28,48,79] | + [51], − [75,79] |  |
| **26** | 3−Methyl−2−butenol | [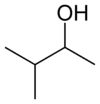](http://en.wikipedia.org/wiki/File:3-methylbutan-2-ol-2D-skeletal.png) | − [79] | − [79] |  | − [79] | − [79] | − [79] |  |
| **27** | Butanol | [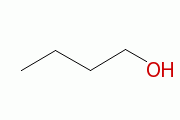](javascript:%20Enlargement('/image?db=1590&id=483535&width=600&height=400',%20600,%20400);) | − [28], + [13,48,75] |  | + [13] | + [28,39,48], − [13,75] | − [28] | + [13,73,74], − [75] | Time dependent |
| **28** | Decanol | [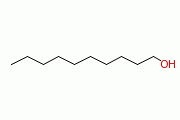](javascript:%20Enlargement('/image?db=1590&id=482707&width=600&height=400',%20600,%20400);) |  |  |  |  |  | + [71] |  |
| **29** | Dodecanol | [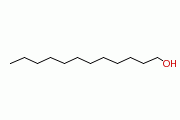](javascript:%20Enlargement('/image?db=1590&id=480010&width=600&height=400',%20600,%20400);) |  |  |  |  |  | + [71] |  |
| **30** | Ethanol | [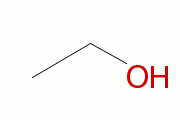](javascript:%20Enlargement('/image?db=1590&id=475123&width=600&height=400',%20600,%20400);) | + [13,48,50,51,56,75,79], − [67,72,77] | + [51,56,72], − [79] | + [13,50], − [77] | + [13,48,49,75,77,79], − [51,56,67,72] | + [77,79] | + [13,49,51,56,67,72,73,74,75,77,79], − [49] | Time dependent |
| **31** | Isobutanol | [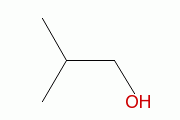](javascript:%20Enlargement('/image?db=1590&id=483537&width=600&height=400',%20600,%20400);) | + [28] |  |  | + [28] | + [28] |  |  |
| **32** | Methanol | [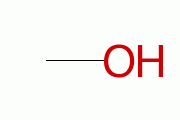](javascript:%20Enlargement('/image?db=1590&id=475176&width=600&height=400',%20600,%20400);) | − [77] |  | − [77] | − [77], + [78] | − [77] | + [73,77] |  |
| **33** | Methylbutanol | 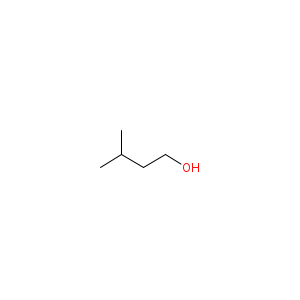 | + [28] |  |  | + [28] | + [28] |  |  |
| **34** | Propanol | [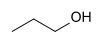](http://upload.wikimedia.org/wikipedia/commons/f/fe/Propan-1-ol.svg) | − [50,51,67], + [50] | − [51] | + [50] | + [49,65], − [49,51,67] |  | + [51,56,65,67], − [49] | Time dependent |
| **35** | Octanol | 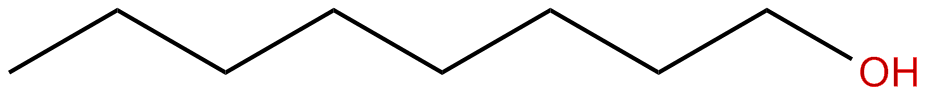 |  |  |  |  |  | + [71] |  |
| **36** | Pentanol | [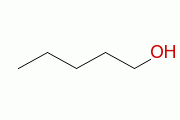](javascript:%20Enlargement('/image?db=1590&id=481385&width=600&height=400',%20600,%20400);) | − [72], + [13] | − [72] | + [13] | − [13,72], + [13] |  | + [13,72] | Time dependent |

Table S3: Volatile acids produced by six pathogenic bacteria

| # |  | Structure | SA | SP | EF | PA | KP | EC | Remarks |
| --- | --- | --- | --- | --- | --- | --- | --- | --- | --- |
| **37** | Acetic acid | [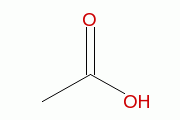](javascript:%20Enlargement('/image?db=1590&id=475727&width=600&height=400',%20600,%20400);) | + [13,48,51,53,56,69,75], − [13,77] | − [51,53,56], + [69] | + [53,69], − [13,77][2, 13] | − [13,51,53,69,77], + [56,69,75] | + [53,69], − [77] | + [13,51,69,73,77] , − [53,75]{Allardyce, 2006 #504} | Time dependent |
| **38** | Butyric acid | [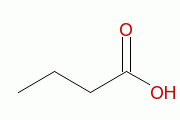](javascript:%20Enlargement('/image?db=1590&id=487089&width=600&height=400',%20600,%20400);) | + [13], − [13,53,69] | − [53,69] | − [53,69] | − [13,53,69] | − [53,69] | − [13,53,69] | Time dependent |
| **39** | Caproic acid | [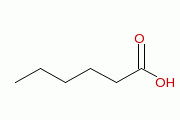](javascript:%20Enlargement('/image?db=1590&id=483343&width=600&height=400',%20600,%20400);) | − [53,69] | − [53,69] | − [53,69] | − [53,69] | − [53,69] | − [53,69] |  |
| **40** | Heptanoic acid | [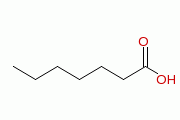](javascript:%20Enlargement('/image?db=1590&id=482229&width=600&height=400',%20600,%20400);) | − [53,69] | − [53,69] | − [53,69] | − [53,69] | − [53,69] | − [53,69] |  |
| **41** | Isobutyric acid | [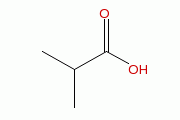](javascript:%20Enlargement('/image?db=1590&id=490870&width=600&height=400',%20600,%20400);) | − [53,69] | − [53,69] | − [53,69] | − [53,69] | − [53,69] | − [53,69] |  |
| **42** | Isovaleric acid | [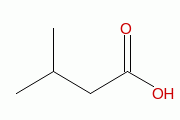](javascript:%20Enlargement('/image?db=1590&id=480375&width=600&height=400',%20600,%20400);) | + [48,53,54,69] | − [53,69] | − [53,69] | − [53,54,69] | + [53], − [69] | − [53,69] |  |
| **43** | Phenylacetic acid | [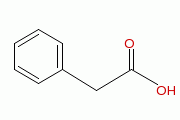](javascript:%20Enlargement('/image?db=1590&id=495300&width=600&height=400',%20600,%20400);) | + [13] |  | + [13] | − [13] |  | + [13] |  |
| **44** | Propionic acid | [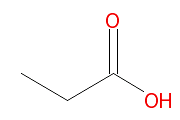](javascript:%20Enlargement('/image?db=1590&id=475328&width=600&height=400',%20600,%20400);) | − [53,69] | − [53,69] | − [53,69] | − [53,69] | + [53,69], − [69] | + [53,69] |  |
| **45** | Valeric acid | [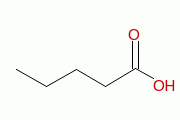](javascript:%20Enlargement('/image?db=1590&id=478562&width=600&height=400',%20600,%20400);) | − [53,69] | − [53,69] | − [53,69] | − [53,69] | − [53,69] | − [53,69] |  |

Table S4: Volatile aldehydes produced by six pathogenic bacteria

| **#** |  | Structure | SA | SP | EF | PA | KP | EC | Remarks |
| --- | --- | --- | --- | --- | --- | --- | --- | --- | --- |
| **46** | 2−Ethyl−propenal | 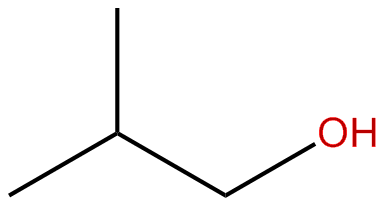 | + [48] |  |  |  |  |  |  |
| **47** | 2−Methyl−2−Butenal | 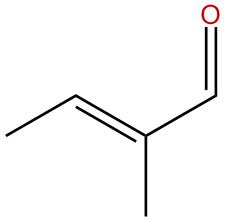 | + [48] |  |  |  |  |  |  |
| **48** | 2−Methyl−butanal | 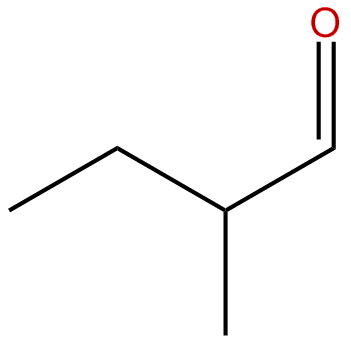 | + [13,28,48,77], − [13] |  | − [13], + [77] | − [13,77], + [28] | − [77], + [28] | − [13,77] | Time dependent |
| **49** | 2−Methyl−propanal | 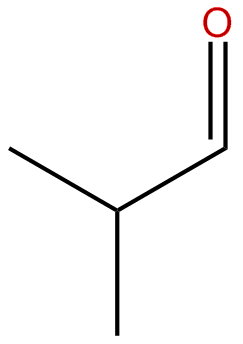 | + [13,48], − [13] |  | − [13] | − [13] |  | − [13] | Time dependent |
| **50** | 2−Methyl−propenal | 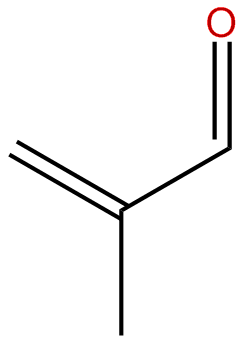 | + [48] |  |  |  |  |  |  |
| **51** | E−2−Octenal | 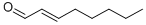 |  |  |  | + [78] |  |  |  |
| **52** | 3−Methyl−2−butenal | 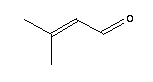 | + [48], − [79] | − [79] |  | − [79] | −[79] | − [79] |  |
| **53** | 3−Methyl−butanal | 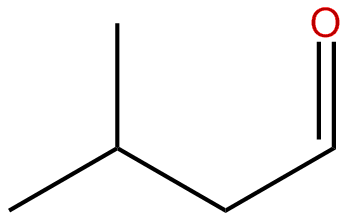 | + [79] | + [79] |  | + [79] | + [79] | + [79] |  |
| **54** | Acetaldehyde | 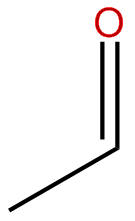 | + [13,48,51,53,56], − [50,72,77] | + [51,53,56,72] | − [50,77], + [50] | − [49,51,56] [56,72,77], + [49] | − [77] | + [51,53,56,72,73], − [49,77] | Time dependent |
| **55** | Benzaldehyde | 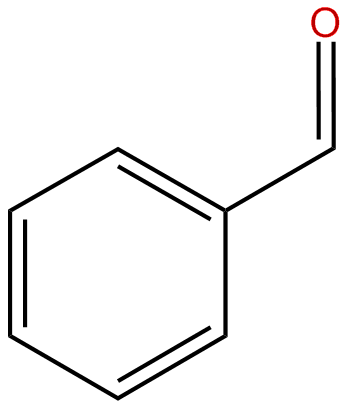 | + [48] |  |  |  |  | + [74] |  |
| **56** | Formaldehyde | 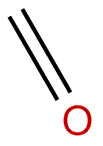 | + [13,50,77], − [50,51] | + [51] | + [13,50,77] | − [51], + [13,49,77] | + [77] | − [51], + [13,49,51,77] | Time dependent |
| **57** | Hexanal | 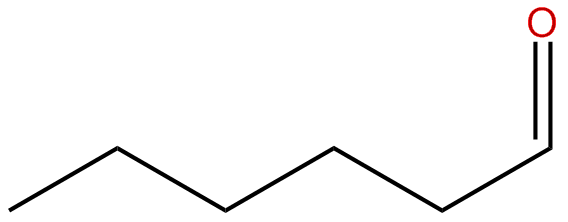 | + [51] | + [51] |  | − [51] |  | + [51] |  |
| **58** | Propanal | 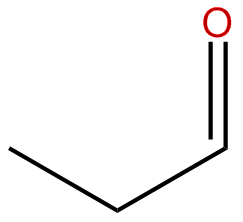 | − [50], + [48] |  | + [50], − [50] | − [49], + [49] |  | − [49] | Time dependent |

Table S5: Volatile ketones produced by six pathogenic bacteria

|  |  | Structure | SA | SP | EF | PA | KP | EC | Remarks |
| --- | --- | --- | --- | --- | --- | --- | --- | --- | --- |
| **59** | 1−Phenyl−1−butanone | 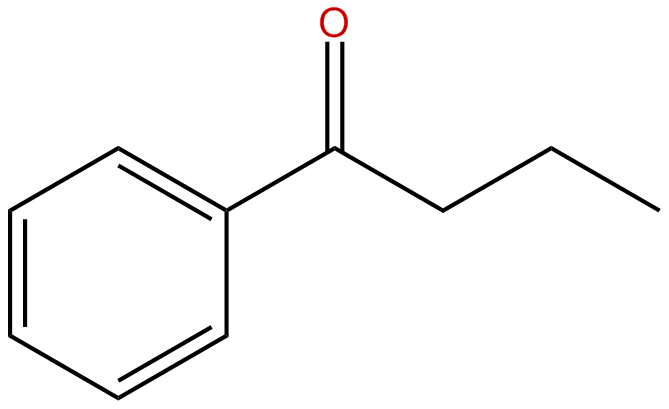 |  |  |  | + [78] |  |  |  |
| **60** | 2,3−Butanedione | 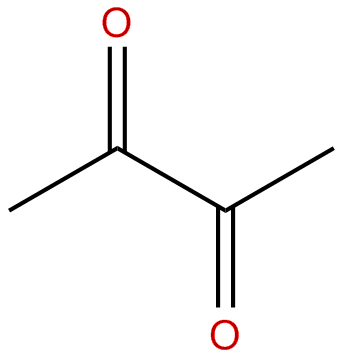 | + [48] |  |  | + [78] |  |  |  |
| **61** | 2,3−Heptanedione | 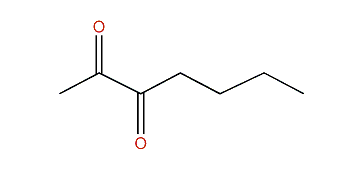 | − [79] | − [79] |  | − [79], + [78] | − [79] | − [79] |  |
| **62** | 2− Nonanone | 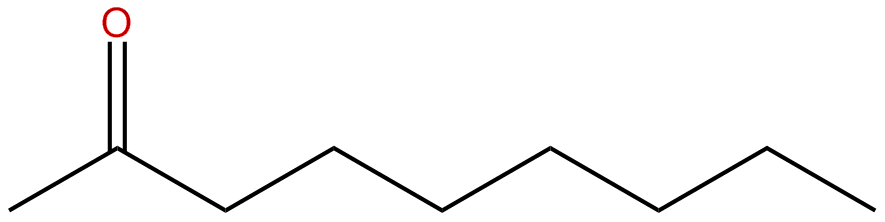 | + [28,75] |  |  | + [39,48,60,78], − [75] | + [28] | + [74], − [75] |  |
| **63** | 2−Butanone | 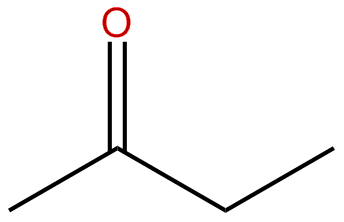 | + [28], − [77] |  | + [77] | + [28,39,48,76], − [77] | + [28], − [77] | + [73], − [77] |  |
| **64** | 2−Dodecanone | 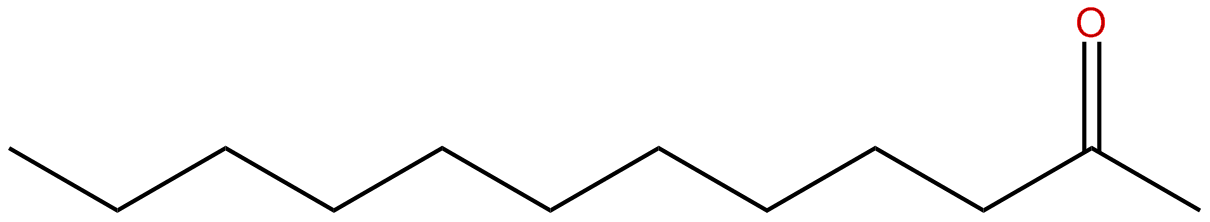 | − [79] | − [79] |  | + [78], − [79] | − [79] | + [79] |  |
| **65** | 2−Hexanone | 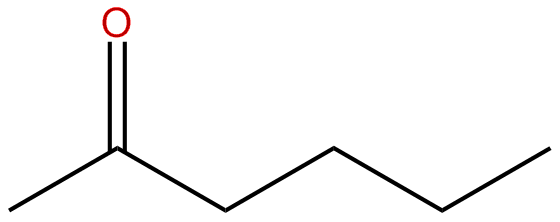 | − [77] |  | − [77] | − [77], + [78] | − [77] | + [74], − [77] |  |
| **66** | 2−Heptanone | 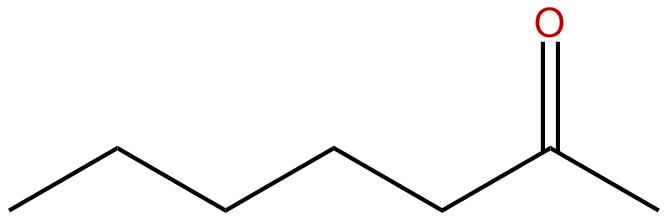 | + [28] |  |  | + [28,48] | + [28] | + [74] |  |
| **67** | 2−Octanone | 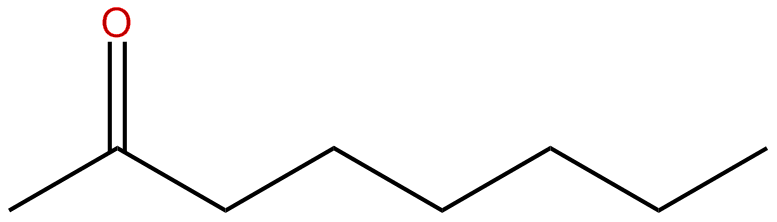 | −[79] | − [79] |  | − [79] | − [79] | − [79] |  |
| **68** | 2−Pentanone | 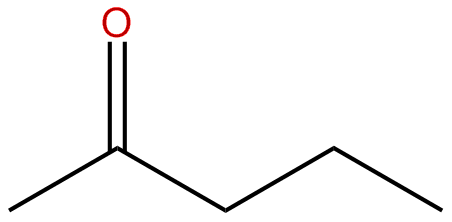 | − [77], + [75] |  | + [77] | − [77], + [48,75,78] | − [77] | − [75,77] |  |
| **69** | 2−Tridecanone | 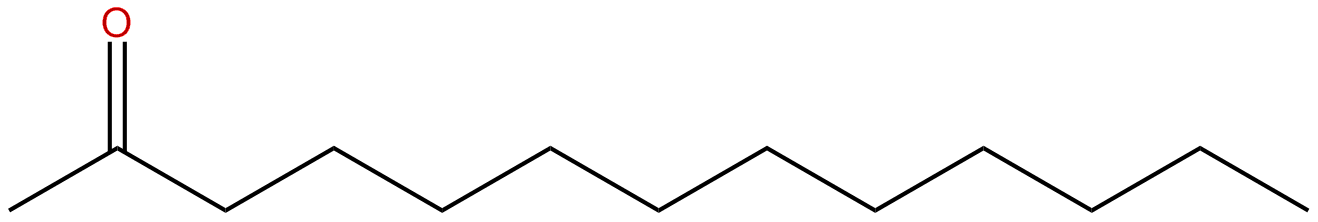 |  |  |  | + [78] |  |  |  |
| **70** | 2−Undecanone | 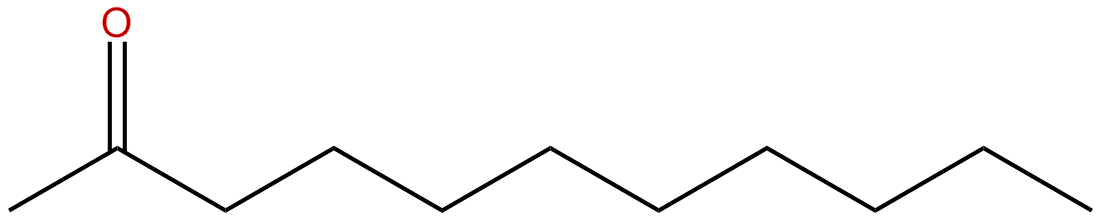 | − [28,79] | − [79] |  | + [39,78], − [79] | + [28], − [79] | − [79] |  |
| **71** | 3−Decanone | 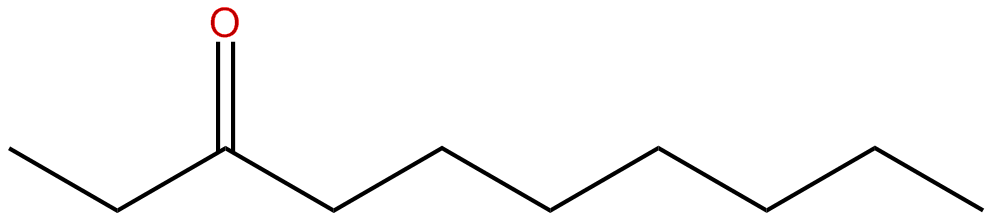 |  |  |  | + [78] |  |  |  |
| **72** | 3−Methyl−2−butanone | 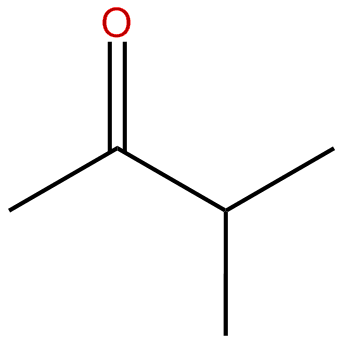 |  |  |  | + [78] |  |  |  |
| **73** | 3−Methyl−2−pentanone | 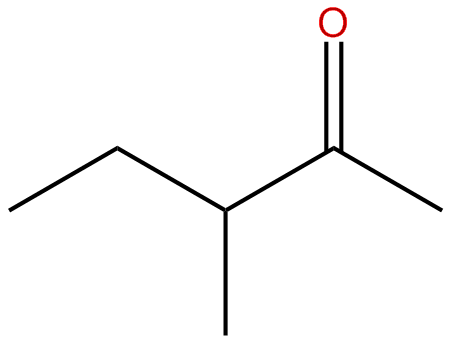 |  |  |  | + [78] |  |  |  |
| **74** | 3−Methyl−3−penten−2−one | 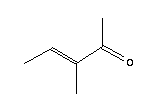 |  |  |  | + [78] |  |  |  |
| **75** | 3−Octanone | 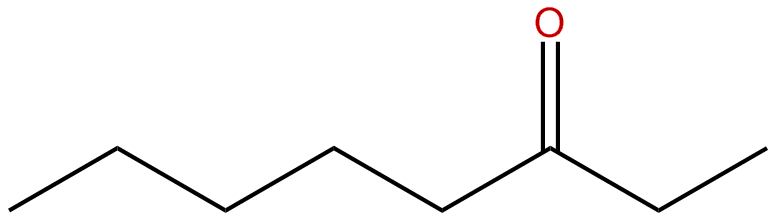 |  |  |  | + [48] |  |  |  |
| **76** | 4−Heptanone | 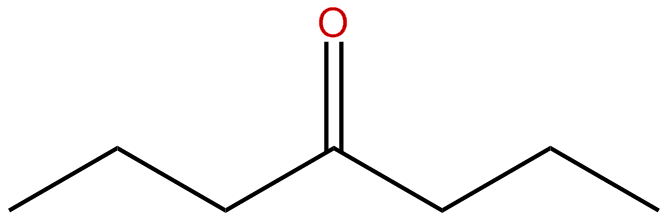 |  |  |  | + [48] |  |  |  |
| **77** | 4−Methyl−2−pentanone | 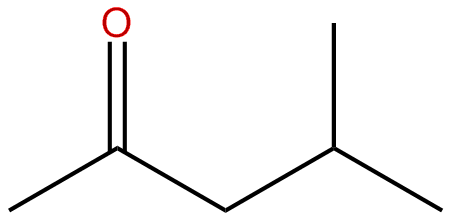 |  |  |  | + [48] |  |  |  |
| **78** | 4−Methyl−4−penten−2−one | 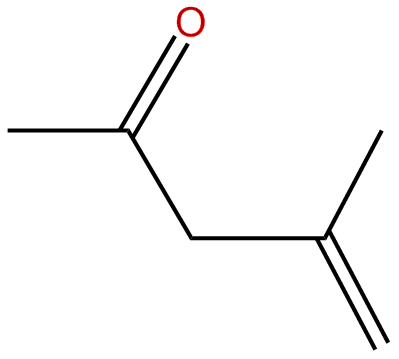 |  |  |  | + [78] |  |  |  |
| **79** | Acetoin | 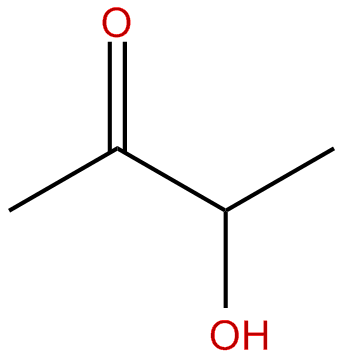 | + [28,48], − [13] |  | − [13] | − [13,28] | − [28] | + [13] |  |
| **80** | Acetol | 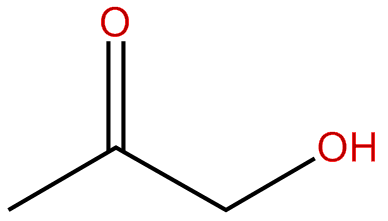 | + [48] |  |  |  |  |  |  |
| **81** | Acetone | 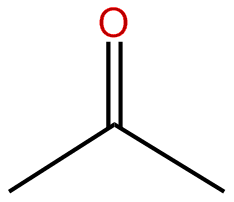 | − [13,50,56,77], + [72,75] | + [53,56,72] | + [13,50,56,77][2, 13], − [13,50] | + [53,56,75,76], − [13,49,72,77] | − [77] | + [13,53,56,72,73,74,77], − [13,49,75] | Time dependent |

Table S6: Cyclic volatile molecules produced by six pathogenic bacteria

| **#** |  | Structure | SA | SP | EF | PA | KP | EC | Remarks |
| --- | --- | --- | --- | --- | --- | --- | --- | --- | --- |
| **82** | 1−Methoxy−4−Methylbenzene | 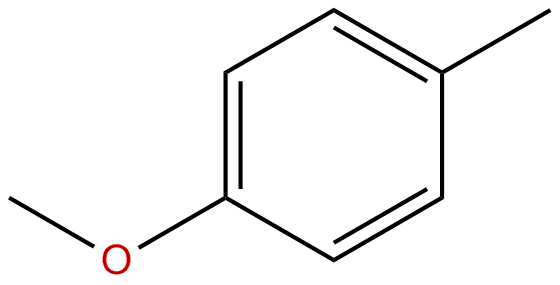 | −[79] | − [79] |  | − [79] | − [79] | − [79] |  |
| **83** | 1−Methyl−naphthalene | 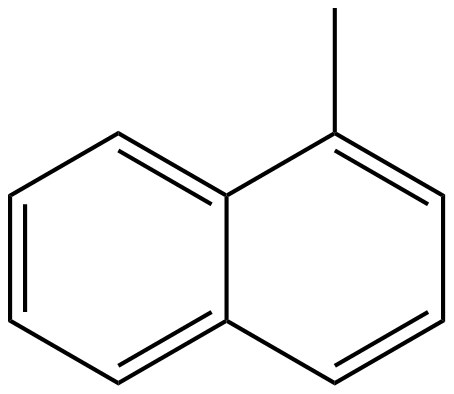 | − [79] | − [79] |  | − [79] | − [79] | + [79] |  |
| **84** | 2−Methyl−naphthalene | 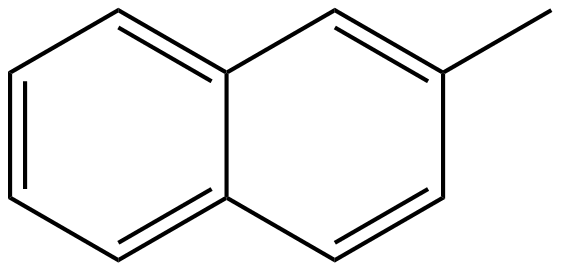 | − [79] | − [79] |  | − [79] | − [79] | + [79] |  |
| **85** | 2−Methyl−phenol | 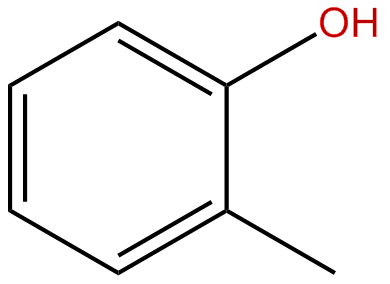 | − [75] |  |  | + [75] |  | + [75] |  |
| **86** | 2−Pentylfuran | 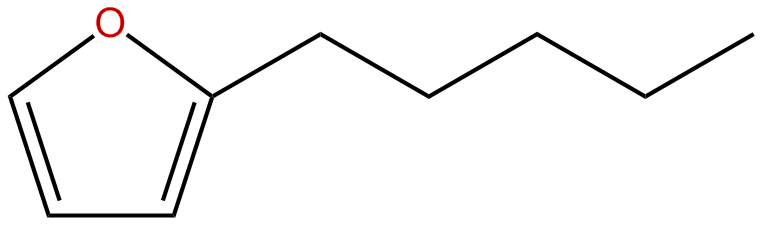 | − [35] | + [35] |  | − [35] |  | − [35] |  |
| **87** | 2−Phenyl ethanol | 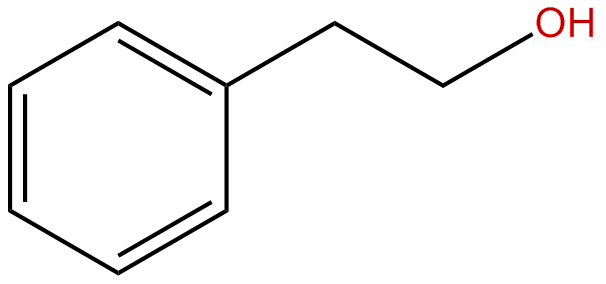 | − [79] | − [79] |  | − [79] | + [79], − [79] | − [79] |  |
| **88** | Acetophenone | 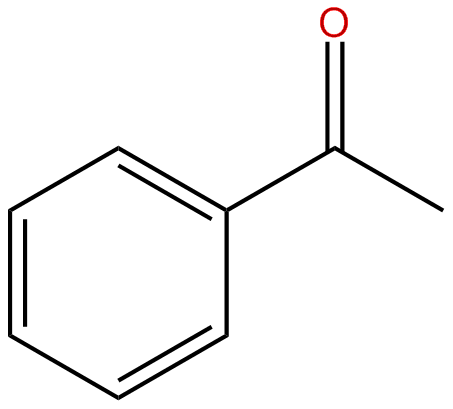 |  |  |  | + [78] |  |  |  |
| **89** | Limonene | 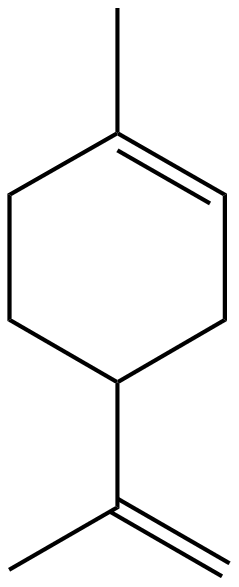 |  |  |  | + [60] |  |  |  |
| **90** | Methyl−benzene−methanol | 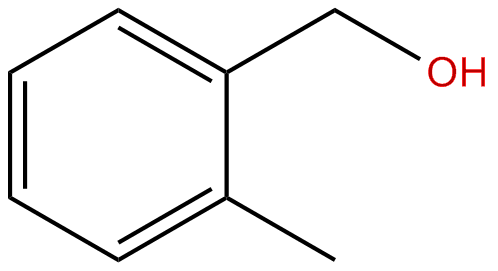 |  |  |  | + [78] |  |  |  |
| **91** | Methyl−benzoate | 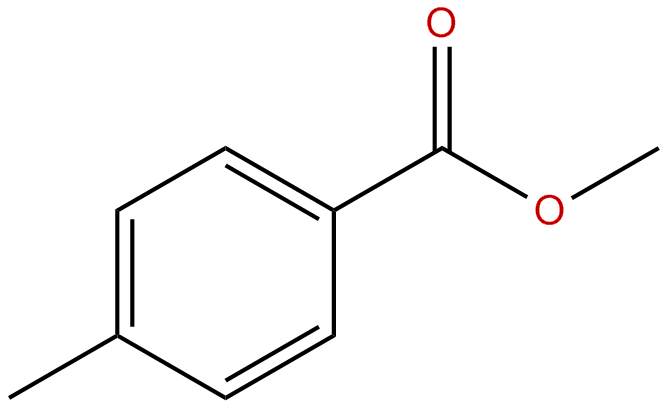 | − [79] | − [79] |  | − [79] | − [79] | − [79] |  |
| **92** | Phenol | 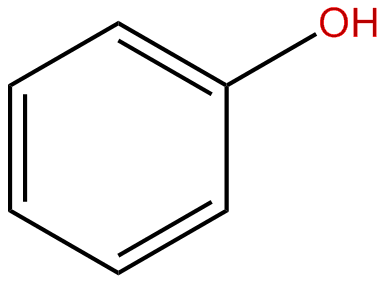 | − [79] | − [79] |  | − [79] | − [79] | + [79] |  |
| **93** | Toluene | 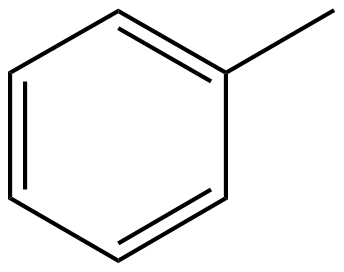 | + [28] |  |  | + [28,39] | + [28] |  |  |

Table S7: Volatile esters produced by six pathogenic bacteria

| **#** |  | Structure | SA | SP | EF | PA | KP | EC | Remarks |
| --- | --- | --- | --- | --- | --- | --- | --- | --- | --- |
| **94** | 2−methylbutyl−isobutyrate | 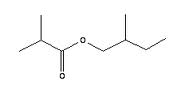 |  |  |  | + [48] |  |  |  |
| **95** | 2−methylbutyl 2−methylbutyrate | 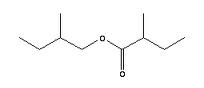 |  |  |  | + [48] |  |  |  |
| **96** | 2−Phenyl−ethyl−acetate | 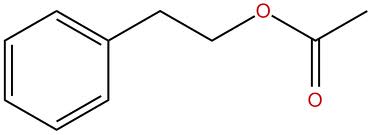 | − [79] | − [79] |  | − [79] | − [79] | − [79] |  |
| **97** | 3−Methylbutyl−2−methyl−propanoate | 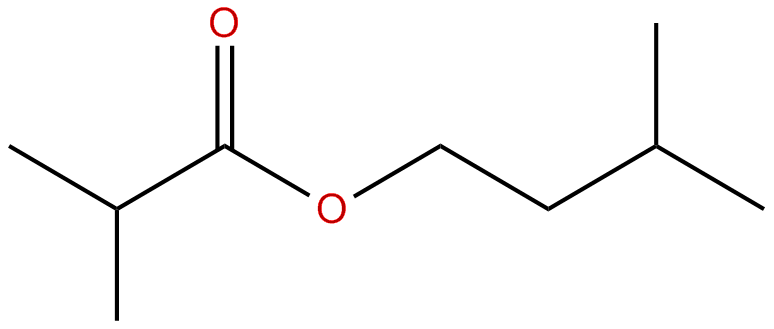 | − [79] | − [79] |  | − [79] | − [79] | − [79] |  |
| **98** | Amylisovalerate | 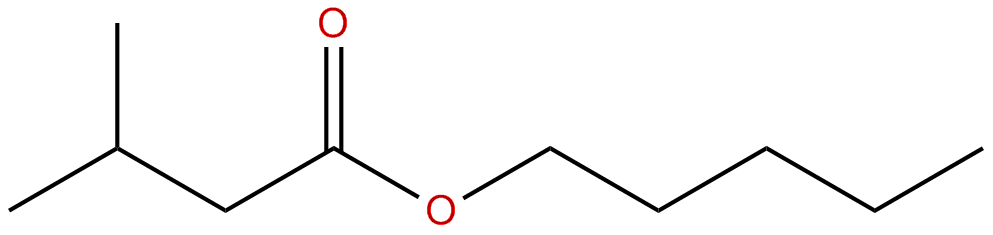 |  |  |  | + [48] |  |  |  |
| **99** | Butyl acetate | 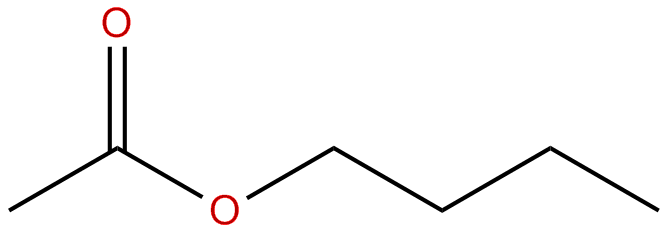 | + [48] |  |  |  |  |  |  |
| **100** | Ethyl acetate | 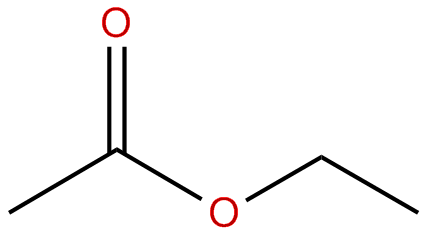 | − [13,67,77], + [48] |  | − [13,77] | − [13,67,77], + [48] | + [13], − [77] | + [13,67,77], |  |
| **101** | Ethyl butanoate |  | + [13], − [77] |  | + [2, 13][77] | + [13], − [77] | + [13], − [77] | + [13,77] |  |
| **102** | Ethyl formate |  | + [48] |  |  |  |  |  |  |
| **103** | Ethyl isovalerate |  | + [48] |  |  |  |  |  |  |
| **104** | Ethyl−2−methyl−butyrate |  |  |  |  | + [48] |  |  |  |
| **105** | Ethyl−phenylacetate |  | − [79] | − [79] |  | − [79] | − [79] | + [79] |  |
| **106** | Isoamyl−benzoate |  | − [79] | − [79] |  | − [79] | − [79] | − [79] |  |
| **107** | Isoamyl−butyrate |  |  |  |  | + [48] |  |  |  |
| **108** | Isopentyl acetate |  | − [28], + [48] |  |  | + [28] | + [28] |  |  |
| **109** | Methyl methacrylate |  | + [48] |  |  | + [48] |  |  |  |
| **110** | Methyl−2−methyl−butyrate |  |  |  |  | + [48] |  |  |  |
| **111** | Propyl acetate |  | − [77] |  | + [77] | − [77] | −[77] | + [77] |  |
| **112** | Phenyl−ethylbutyrate |  | − [79] | − [79] |  | − [79] | − [79] | − [79] |  |
| **113** | Phenylacetic acid propylester |  | − [79] | − [79] |  | − [79] | − [79] | + [79] |  |

Table S8: S−containing volatile molecules produced by six pathogenic bacteria

| **#** |  | Structure | SA | SP | EF | PA | KP | EC | Remarks |
| --- | --- | --- | --- | --- | --- | --- | --- | --- | --- |
| **114** | 2−(Methylthio)−ethanol |  | + [79] | − [79] |  | − [79] | + [79] | + [79] |  |
| **115** | 2−Methoxy−5−methyl thiophene |  |  |  |  | + [48] |  |  |  |
| **116** | 3−(Ethylthio)−propanal |  |  |  |  | + [48] |  |  |  |
| **117** | DMDS |  | − [13,54,56,77,79] + [13,28,48] | − [56,79] | + [77], − [13] | + [13,28,39,48,54,56,68,70,76,79], − [77] | + [13,28,77], − [79] | + [13,51,77,79], − [13] | Time dependent |
| **118** | DMS |  | + [56], − [54,72,77] | + [56,72] | + [77] | + [48,54,56,76], − [72,77] |  | + [56,77], − [72] | Time dependent |
| **119** | DMTS |  | − [79] | − [79] |  | + [4, 9][48,68], − [79] | − [79] | + [79] |  |
| **120** | Hydrogen−sulfide |  | + [13,56], − [50,72,77] | − [56], + [72] | + [13,50,77], − [50] | − [49,56,72,77], + [13] | + [77] | − [49,56], + [13,49,72,77] | Time dependent |
| **121** | Mercaptoacetone |  |  |  |  | + [48] |  |  |  |
| **122** | Methyl−mercaptan |  | − [72], + [13,48,56,77] | − [56], + [72] | + [13,77] | − [56,72], + [13,48,70,77] | + [77] | + [13,56,72,73,77] |  |
| **123** | Methyl−thioacetate |  |  |  |  | + [78] |  |  |  |
| **124** | S−Methyl−thiobenzoate |  | − [79] | − [79] |  | − [79] | − [79] | − [79] |  |

Table S9: N−containing volatile molecules produced by six pathogenic bacteria

| **#** |  | Structure | SA | SP | EF | PA | KP | EC | Remarks |
| --- | --- | --- | --- | --- | --- | --- | --- | --- | --- |
| **125** | 1−Vinyl aziridine |  |  |  |  | + [48] |  |  |  |
| **126** | 2,3,5−Trimethyl−pyrazine |  | − [79] | − [79] |  | − [79] | − [79] | + [79] |  |
| **127** | 2,4−dimethyl−quinazoline |  |  |  |  | + [66] |  |  |  |
| **128** | 2,5−Dimethyl−pyrazine |  |  |  |  | + [78] |  |  |  |
| **129** | 2−(3−Methylbutyl)−3,5−dimethyl−pyrazine |  | − [79] | − [79] |  | + [79] | − [79] | − [79] |  |
| **130** | 2−AA |  | + [13,51], − [13,54,72,77,79] | + [51,72], − [79] | + [13], − [77] | + [13,39,40,54,66,78,79], − [13,51,72,77] | − [77], + [79] | + [13,51,72,77,79] − [66] | Time dependent |
| **131** | 2−Acetylthiazole |  | − [79] | − [79] |  | − [79] | − [79] | − [79] |  |
| **132** | 2−Benzoxazole |  |  |  |  | + [78] |  |  |  |
| **133** | 2−Ethyl−3−(methylthio)−pyrazine |  |  |  |  | + [78] |  |  |  |
| **134** | 2−Methyl−3−isopropyl−pyrazine |  |  |  |  | + [78] |  |  |  |
| **135** | 3−Methyl−1H−indole |  | − [79] | − [79] |  | − [79] | − [79] | + [79] |  |
| **136** | 3−Methyl−N−(2−phneylethylidene)−1−Butanamine |  | − [79] | − [79] |  | − [79] | − [79] | − [79] |  |
| **137** | 3−Methyl−N−(3−methylbutylidene)−1−butanamine |  | − [79] | − [79] |  | − [79] | − [79] | − [79] |  |
| **138** | 3−Methylpyrrole |  |  |  |  | + [48] |  |  |  |
| **139** | 4−Chloro−1H−indole |  | − [79] | − [79] |  | − [79] | − [79] | + [79] |  |
| **140** | 4−Methyl−quinazoline |  |  |  |  | + [66,78] |  |  |  |
| **141** | Acetonitrile |  | + [75] |  |  | + [70,75] |  | + [75] |  |
| **142** | Ammonia |  | + [50,56,72,77], − [13] | − [56,72] | − [13], + [50,77] | + [13,49,70,77], − [56,72] | + [77] | − [13,56,72,77], + [49] |  |
| **143** | Benzonitrile |  | − [79] | + [79] |  | + [78,79] | − [79] | + [79] |  |
| **144** | Benzoxazole |  |  |  |  | + [78] |  |  |  |
| **145** | Benzoxazole |  |  |  |  | + [78] |  |  |  |
| **146** | Benzyl−nitrile |  | − [79] | X [79] |  | − [79] | − [79] | − [79] |  |
| **147** | Hydrogen−cyanide |  |  |  |  | + [48,52,70,76] |  |  |  |
| **148** | Indole |  | + [51], − [13,72,75,77,79] | + [51], − [72,79] | − [13,72,77] | − [13,51,72,77,79], + [13,75] | − [79] | + [13,51,72,73,75,77,79] | Time dependent |
| **149** | Methyl pyrazine |  | − [79] | − [79] |  | + [78], −[79] | − [79] | + [79] |  |
| **150** | Methyl−thiocyanide |  |  |  |  | + [48,76] |  |  |  |
| **151** | N,N′−Dibenzylideneethylenediamine |  | − [79] | − [79] |  | − [79] | − [79] | + [79] |  |
| **152** | N−(1,1−Dimethylethyl)−benzamide |  | − [79] | − [79] |  | − [79] | − [79] | − [79] |  |
| **153** | N−Butyl−benzenamine |  | − [79] | − [79] |  | − [79] | − [79] | − [79] |  |
| **154** | N−n−Butylphthalimide |  | − [79] | − [79] |  | − [79] | − [79] | − [79] |  |
| **155** | N−Phenyl−methylene−1−butanamine |  | −[79] | − [79] |  | − [79] | − [79] | + [79] |  |
| **156** | N−Phenyl−methylene−1−propanamine |  | − [79] | − [79] |  | − [79] | − [79] | + [79] |  |
| **157** | N−Phenyl−methylene−methanamine |  | − [79] | − [79] |  | − [79] | − [79] | + [79] |  |
| **158** | p−Pentylaniline |  | − [79] | − [79] |  | − [79] | − [79] | − [79] |  |
| **159** | Pyrimidine |  | + [75] |  |  | − [75] |  | − [75] |  |
| **160** | Pyrrole |  | − [13], + [13] |  | + [13] | − [13], + [13,48] |  | + [13], − [13] | Time dependent |
| **161** | Trimethyl−amine |  | + [13,51], − [13,67,77] | + [51] | + [13], − [77] | − [51,67,77], + [13] | + [77] | + [13,51,67,77] | Time dependent |
